# Probiotic-Fermented Camel Milk Attenuates Neurodegenerative Symptoms via SOX5/miR-218 Axis Orchestration in Mouse Models

**DOI:** 10.3390/ph16030357

**Published:** 2023-02-25

**Authors:** Ashraf Khalifa, Hairul Islam Mohamed Ibrahim, Abdullah Sheikh, Hany Ezzat Khalil

**Affiliations:** 1 Biological Science Department, College of Science, King Faisal University, P.O. Box 400, Al-Ahsa 31982, Saudi Arabia; 2 Botany and Microbiology Department, Faculty of Science, Beni-Suef University, Beni-Suef 62511, Egypt; 3 Molecular Biology Division, Pondicherry Centre for Biological Sciences and Educational Trust, Pondicherry 605004, India; 4 Camel Research Center, King Faisal University, P.O. Box 400, Al-Ahsa 31982, Saudi Arabia; 5 Department of Pharmaceutical Sciences, College of Clinical Pharmacy, King Faisal University, Al-Ahsa 31982, Saudi Arabia; 6 Department of Pharmacognosy, Faculty of Pharmacy, Minia University, Minia 61519, Egypt

**Keywords:** *Bacillus*, fermented camel’s milk, autoimmune disorder, myelin-binding protein, SOX5, multiple sclerosis

## Abstract

Multiple sclerosis is an autoimmune-mediated myelin damage disorder in the central nervous system that is widespread among neurological patients. It has been demonstrated that several genetic and epigenetic factors control autoimmune encephalomyelitis (EAE), a murine model of MS, through CD4+ T-cell population quantity. Alterations in the gut microbiota influence neuroprotectiveness via unexplored mechanisms. In this study, the ameliorative effect of *Bacillus amyloliquefaciens* fermented in camel milk (BEY) on an autoimmune-mediated neurodegenerative model using myelin oligodendrocyte glycoprotein/complete fraud adjuvant/pertussis toxin (MCP)-immunized C57BL6j mice is investigated. Anti-inflammatory activity was confirmed in the in vitro cell model, and inflammatory cytokines interleukins IL17 (from EAE 311 to BEY 227 pg/mL), IL6 (from EAE 103 to BEY 65 pg/mL), IFNγ (from EAE 423 to BEY 243 pg/mL) and TGFβ (from EAE 74 to BEY 133 pg/mL) were significantly reduced in BEY-treated mice. The epigenetic factor miR-218-5P was identified and confirmed its mRNA target SOX-5 using in silico tools and expression techniques, suggesting SOX5/miR-218-5p could serve as an exclusive diagnostic marker for MS. Furthermore, BEY improved the short-chain fatty acids, in particular butyrate (from 0.57 to 0.85 µM) and caproic (from 0.64 to 1.33 µM) acids, in the MCP mouse group. BEY treatment significantly regulated the expression of inflammatory transcripts in EAE mice and upregulated neuroprotective markers such as neurexin (from 0.65- to 1.22-fold) (*p* < 0.05), vascular endothelial adhesion molecules (from 0.41- to 0.76-fold) and myelin-binding protein (from 0.46- to 0.89-fold) (*p* < 0.03). These findings suggest that BEY could be a promising clinical approach for the curative treatment of neurodegenerative diseases and could promote the use of probiotic food as medicine.

## 1. Introduction

Recently, extensive studies have been conducted on the effect of fermented food products on human health, including probiotics (PBT) and fermented dairy products, as either prophylactic or therapeutic nutraceuticals [1]. PBTs are considered one of the food supplements that are produced using non-pathogenic microorganisms that improve and restore gut commensal microbiomes (GMs) [2]. Reports evidenced that PBTs could potentially improve a variety of gastrointestinal disorders and improve health [3]. Daily consumption of PBTs demonstrated a modulation of the immune system, leading to improvements in health, including a reduction in the complications of multiple sclerosis (MS) [3,4,5]. PBT bacteria include *Lactobacilli, Bacillus, Bifidobacteria and Enterococci* [6]. Several studies have shown the ameliorative properties of PBTs as food supplements via the regulation of imbalanced GM [7]. Moreover, PBTs have demonstrated alleviation of immuno-inflammatory issues associated with multiple health conditions, including diabetes, inflammatory bowel syndrome (IBS), neuro-inflammatory illnesses and MS [2,8,9,10]. Currently, live PBT cultures are part of fermented dairy products. Milk, including camel milk (CM), is rich in Lactic acid bacteria and *Bifidobacteria*, which makes it a perfect medium for the growth of PBTs [11]. CM is a natural endowment that is gifted with very beneficial effects on human health by maintaining the appropriate functionality of the gastrointestinal mucosa and toning the inflammatory mediators in the gut [12]. CM has demonstrated a unique composition by being rich in essential contents for the growth of PBTs and helping them to produce a plethora of biologically active metabolites [13]. Moreover, fresh CM has alleviated the pro-inflammatory and necrotic mediators at the level of the gut [14]. Fermented CM is presumed to have exceptional nutraceutical and immunomodulatory characteristics due to the presence of a significant diversity of bioactive metabolites, including beneficial health-promoting peptides and bacteria, when compared with fresh CM [13,15]. MS is a complex neurological syndrome leading to inflammation as well as demyelination of the central nervous system, accompanied by different levels of injury to neurons [16]. The exact pathological events associated with the development of MS remain unclear. However, the MS pathology cascade could be attributed to multiple factors, including damage to and/or loss of oligodendrocytes and neurons [17,18,19]. Demyelination associated with axonal injury is considered one of the most prominent causes of neurologically related MS that could lead to certain limitations in disability [20]. Consequently, the discovery of new medical strategies with immunomodulatory and anti-inflammatory potential could be helpful to control MS. GM consists of colonies of microorganisms forming a complex mutually dynamic system in the human gut that plays essential metabolic and immune regulatory roles [21]. GM–host interactions have demonstrated axial correlations between various body systems and the brain [22,23]. Interestingly, GM is able to support the host with bio-metabolites that trigger immune and inflammatory responses through their communications with the central nervous system, which was evidenced via the secretion of bio-polysaccharides by *Bacillus fragilis*, indicating intestinal mucosa/brain-associated interaction [24]. Moreover, GM can regulate the release of cellular cytokines and chemokines. Accordingly, GM may exhibit a characteristic promoting action on the release of host neuro-mediators. This type of distinct mutual loop between GM and the host could indirectly induce an immune-regulatory association and result in the expression of neuroactive and bio-amine mediators by the host [25]. Consequently, this signaling pathway will improve the impairments associated with MS [26]. Studies have demonstrated that SCFAs (short-chain fatty acids) are presumed to modulate host immunity and reduce inflammation, leading to an improvement in colon health [27,28,29]. GM is considered one of the substantial initiators of the fermentation process that is required to convert indigestible carbohydrates into SCFAs. Studies have also reported GM-immune system-associated mechanisms with various modulations of the host microRNAs in an animal model of MS and experimental autoimmune encephalomyelitis (EAE) [30,31,32]. In addition, the genomic interactions between GM and host have not been fully explored; hence, the associated molecular patterns need to be elucidated [33]. The current approach highlights the importance of fermented dairy products as a source of PBTs. Based on the fact that fermented CM is reputed to contain a variety of bioactive nutrients that have demonstrated many biological activities [34], the conducted preventive study highlighted the importance of *Bacillus amyloliquefaciens* (BA) enriched with CM on the expression of pro-inflammatory cytokines in induced IBS in a mouse model [12]. Interestingly, fermented CM supplemented with BA (BEY) serves as a PBT dairy product and could be a therapy for the cure of MS-associated disorders. Therefore, based on the concept that the regulation of GM can play a crucial role in MS pathophysiology, it is beneficial to explore and elucidate the possible modulatory mechanisms exerted by BEY to cure the symptoms associated with MS. To evaluate the potential curative strategy to manage dysfunctions accompanied by MS, this study was conducted using an MCP-immunized C57BL6j mouse model. Specifically, the pattern of inflammatory markers, immunoblot, qPCR, pathological scores and severity of the disease in treated and untreated mice were assessed.

## 2. Results

In our previous work, we demonstrated that the sensory quality of BEY was significantly improved compared to traditional cow’s milk yogurt. For this reason, BEY was further investigated as a curative PBT formulation for EAE.

### 2.1. The Effect of BEY on SOX5 Regulation in MOG-Induced Splenocytes

**Figure 1:**
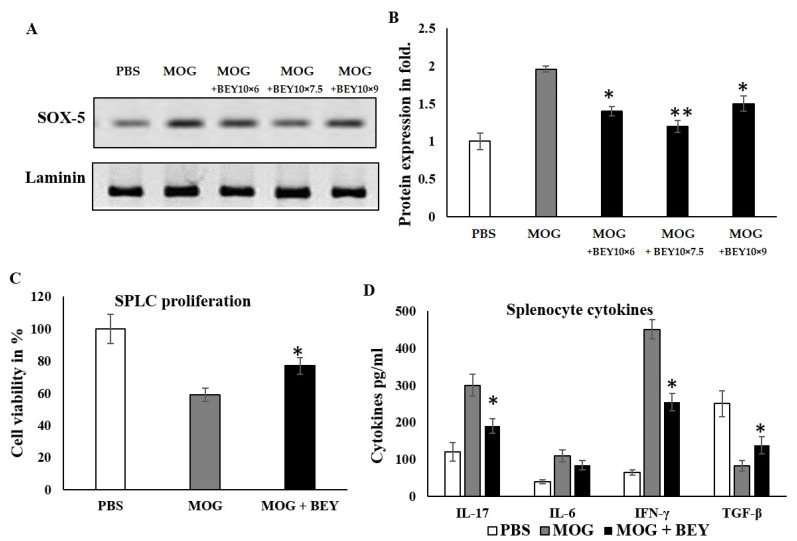
The effect of BEY on MOG-stimulated splenic lymphocytes. (**A**,**B**) Total lymphocytes were × collected from naïve mouse spleens and immunized with 2 ng/mL MOG for 4 h and treated with BEY for 2 h. Treated cells were evaluated for SOX5 transcriptional factors using Western blot. (**C**,**D**) The optimum BA for yogurt is 10 × 7.5, which was further used in splenocyte viability testing with the SRB assay. The level of cytokine secretion in splenocytes was quantified using ELISA kit. The values were expressed as pg/mL and each group’s data were displayed as mean ± SD. One-way ANOVA followed by post hoc *t*-test was used for statistical analysis (significant at * *p* ≤0.05, ** *p* ≤ 0.01).

Naïve mouse spleens were excised and mononuclear cells were isolated and cultured for 4 h in the presence of 2 ng/mL of myelin oligodendrocyte glycoprotein (MOG) 35–55. Transcriptional factors regulate the polarization of inflammatory cell differentiation and maturation. In this study, the effect of BEY on the regulation of SRY-box transcription factor 5 (SOX5) in MOG-induced splenocytes was examined. The data are presented in Figure 1. As can be seen in Figure 1A,B, SOX-5 facilitates the looping between lymphoid gene promoters to promote EAE pathogenesis and Th17 differentiation. BEY-treated splenocytes showed a significant reduction (from 2.03- to 1.3-fold) in SOX5 protein expression compared to MOG-treated cells at 10 *×* 7.5 cfu of BA-inoculated BEY. Furthermore, the proliferation level of BEY-treated splenocytes was substantially increased compared to that of MOG-treated cells (Figure 1C). MOG-stimulated splenocytes enhanced the expression levels of inflammatory cytokines, whereas the BEY treatment significantly reduced these inflammatory markers (*p* < 0.04). In addition, the level of interleukin-17 (IL-17) and IFN-γ released from Th17 cells was remarkably decreased in the BEY-treated cells (Figure 1D). Expectedly, the reciprocal regulation of interferon gamma (IFN-γ) and transforming growth factor beta (TGF-β) was noted in BEY-treated MOG-challenged splenocytes (Figure 1D). However, the interleukin-6 (IL-6) cytokine in the BEY-treated group exhibited an insignificant change compared to the MOG-treated cells.

### 2.2. Upregulation of the Epigenetic Factors (miRNA218-5p) by BEY Treatment

**Figure 2:**
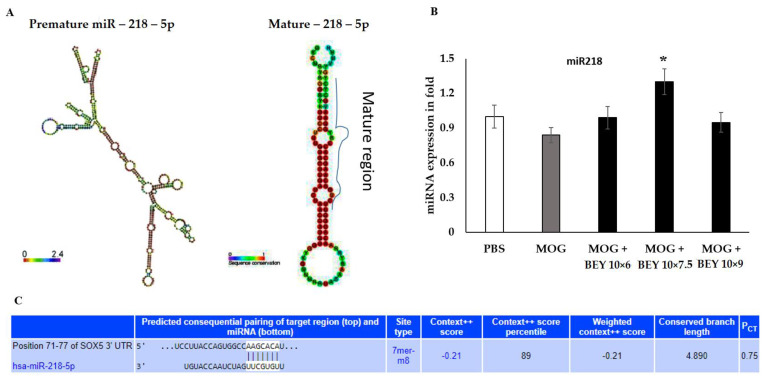
Effect of BEY on epigenetic hindrance of SOX5 expression. (**A**) SOX5 predominant miRNA target miR218 was identified using target scanning. (**B**) The expression of miR218-5p was quantified using Real-Time PCR. The results were reciprocally correlated with SOX5 protein expression. (**C**) miR-218-5p targets mice SOX5 3’ UTR regions from 71-77 basepair and it was confirmed by *insilico* target scan tool. Values are expressed using Student’s *t*-test and one-way ANOVA (significant at * *p* ≤ 0.05).

SOX5 expression was confirmed in splenocytes, and a set of SOX5 miRNA inhibitors (miR132, miR21 and miR218) was selected based on target scan tools. The virtual binding of miRNAs was selected for further molecular analysis. To validate and calibrate the expression levels of randomly chosen miRNAs along with the U6 internal control, they were analyzed in qRT-PCR (quantitative Real-Time PCR). The miRNAs were defined by a premature and mature similarity index, with fewer loop and hairpin nucleotides exposed in Figure 2A. The expression of miRNA 218-5p was evaluated, and we found that the BEY (10 Log 7.5) inoculum significantly upregulated miR218 in MCP-stimulated cells (Figure 2B). The promoter region of SOX5 at 71–7 base pairs complexed with miR-218 was confirmed (shown in Figure 2C). Taken together, the results of miR218 validation using qRT-PCR were confirmed in further experiments.

### 2.3. Pathology of EAE C57BL6j Mice Treated with BEY

**Figure 3:**
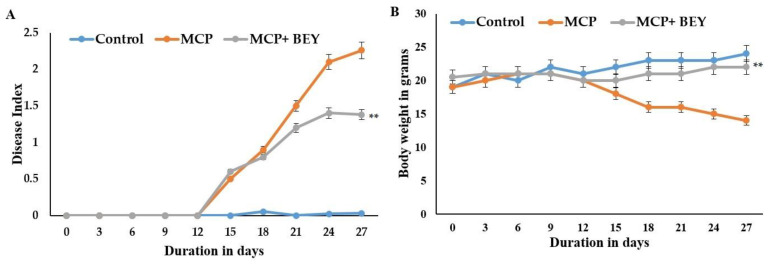
Effect of BEY on in vivo EAE in C57Bl/6J mouse model. Mice were immunized with MOG and pertussis toxin on 1st and 3rd days, respectively. Beginning on the third day of immunization, mice were given BEY (10 × 7.5 cfu load/20 g of mouse body weight). (**A**) Disease index of EAE pathology over 27 days of MOG immunization. The clinical signs were graded as described in the Materials and methods section; (**B**) body weight (g) of EAE mice and BEY-treated mice. Values are expressed using Student’s *t*-test and one-way ANOVA (significant at ** *p* ≤ 0.01).

Clinical scores and disease indicators were used to quantify the degree to which physiological symptoms represented a recovery in the MS model. In this investigation, an in vivo mouse model of multiple sclerosis was used to assess whether BEY supplementation could reverse the demyelination and paralysis signs seen in these animals. EAE-disease mice displayed severe MS clinical signs, including tail and hind limb paralysis, after 15 days of MCP immunization. The symptoms peaked after the 18*th* day and persisted until the end of the experiment (Figure 3A). The treatment of EAE mice with BEY resulted in significant symptom recovery and attenuated the paralysis symptoms after the 20th day of MCP immunization. After the 24th day of observation, the clinical symptoms were potentially reduced with BEY treatment. These results were further confirmed through body weight measurements, tissue pathological features and cytokine expression levels. BEY improved the body weight of EAE mice from 14 to 22 g (Figure 3B).

### 2.4. The Positive Effect of BEY on EAE Pathology in C57Bl6j Mice

**Figure 4:**
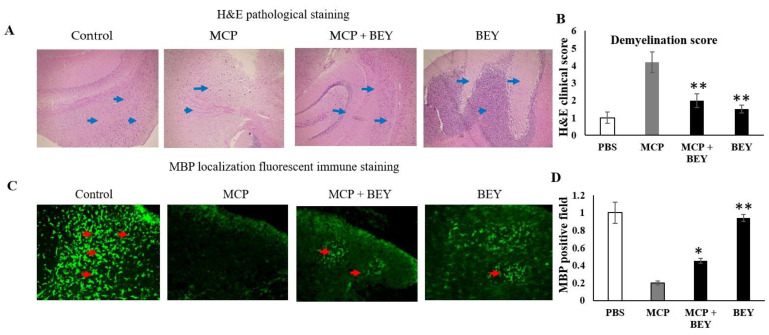
BEY augmented the histopathological condition in the CNS of EAE mice. On the 27th day of MOG immunization, EAE mice were sacrificed, and their spinal cords were extracted and fixed in formaldehyde (10%), followed by paraffin embedding and preparation of 4 µm-thick sections. (**A**) Spinal cords were assessed for demyelination using H&E, and the pathological parameters were noted at 200X resolution. (**B**) Demyelination, infiltration and inflammation scores were recorded. (**C**,**D**) Immunofluorescence quantification of MBP to evaluate myelin sheath damage after 16 days of MOG induction. MBP is indicated by a green fluorescence. The myelin damage in the subcortical white matter of spinal cord was alleviated in BEY group at 27 days of MOG induction. We repeated three independent experiments to extract the data and represented the results as mean ± SD; * significant at * *p* ≤ 0.05, ** *p* ≤ 0.01.

BEY improved the histopathological conditions in the CNS of EAE mice (Figure 4). The histopathological observations of the peripheral nervous system (white and gray matter), the myelin damage (from 3.92-fold to 2.1-fold) and the T-cell infiltration were obviously reduced by the administration of BEY to the MCP-immunized animal model (Figure 4A). Moreover, the inflammatory damage level (*p* < 0.03) was significantly decreased and the pathophysiology score was alleviated in BEY-treated mice. Interestingly, a substantial decrease in neutrophil infiltration was observed in the spinal cord of BEY-treated EAE mice (Figure 4B). Furthermore, the immunofluorescence score of the myelin-binding protein was markedly increased (from 0.18- to 0.42-fold) in EAE mice treated with BEY (Figure 4C,D). Therefore, BEY treatment improved the kinetic function and ameliorated the infiltration and oxidative damage of the myelin sheath in the spinal cords of EAE mice.

### 2.5. Effect of BEY on Biochemical and Inflammatory Markers in MCP-Induced EAE Mice

**Figure 5:**
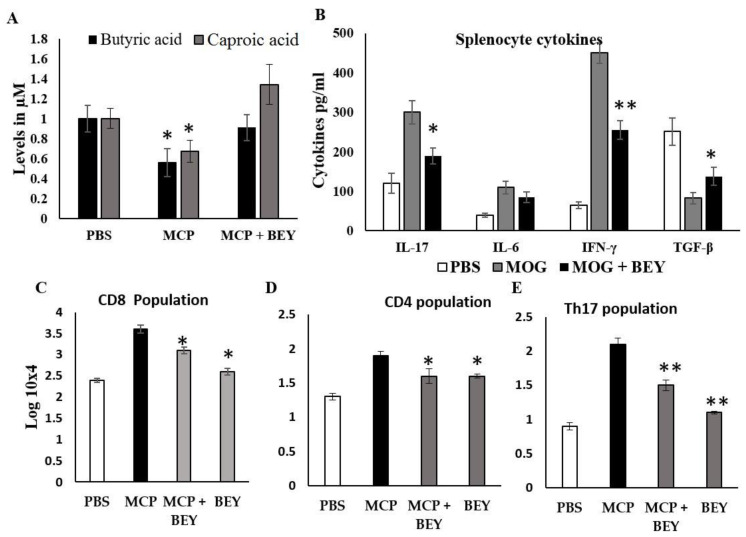
Effect of BEY on biochemical and inflammatory modulators in MOG-induced EAE mice. The splenic cells from naïve mice were examined for SCFAs (short-chain fatty acids), cytokines and T-cell inflammatory cell population in spinal cord. (**A**) Butyric acid and caproic acid were quantified, and values are expressed in µM. (**B**) The expression levels of cytokines (IL-17, IL-6, IFN-γ and TGF-β) were estimated using ELISA Kit (Invitrogen, CA, USA; Biovision, Abcam, USA). (**C**–**E**). CD4, CD8 and Th17 cell populations were counted in spinal cord of BEY-treated EAE mice. For each group, values were expressed as mean ± SD. For statistical analysis, one-way ANOVA was used, followed by a post hoc *t*-test (significant at * *p* ≤ 0.05, ** *p* ≤ 0.01).

**Figure 6:**
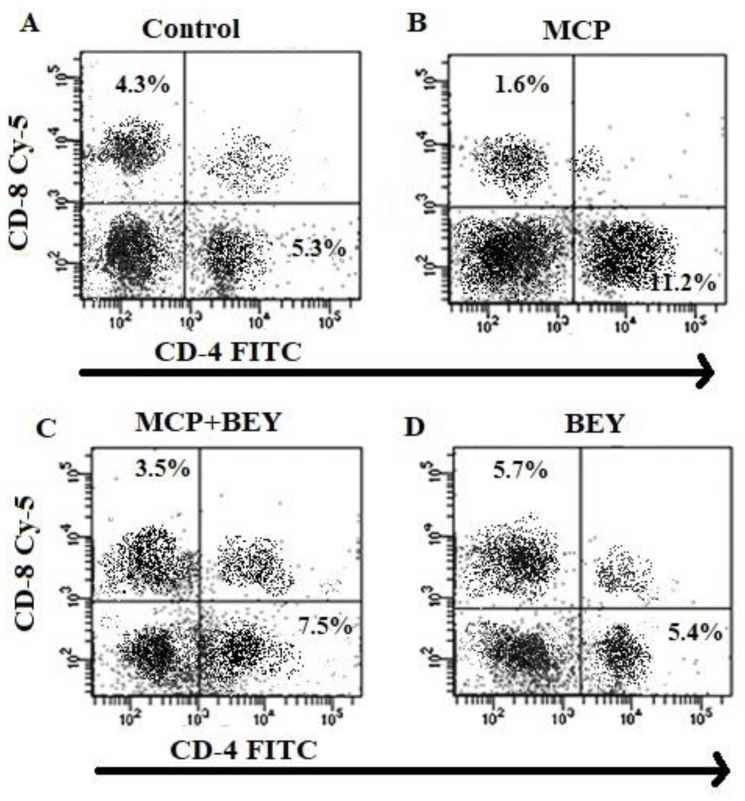
Flow cytometric analysis of T-cell population of EAE spleen was controlled by BEY supplementation. Reactivation of cell suspensions from the pooled spleen was carried out using MOG (10 μg/mL) for 6 h. The frequencies of CD4^+^ and CD4^+^ were analyzed by FACS. (**A**) Control group cells. Representative FACS dots represented the frequency of CD4^+^ and CD4^+^ by CD65L differentiation in each group. The percentage of CD4^+^ and CD4^+^ T cells in each group was determined. (**B**) MCP-treated cells. (**C**). MCP-BEY-treated group. (**D**) BEY-alone-treated group. The data are representative of three independent experiments.

The effect of BEY on the SCFAs of EAE mice was quantified, and the data are displayed in Figure 5A. Two SCFAs, butyric and caproic acids, were substantially upregulated in BEY-treated mice compared to the other EAE group (Figure 5A). The level of butyric acid was increased from 0.55 μM to 0.95 μM in BEY-treated spinal cord tissue. A similar pattern was observed in the caproic acid levels. These results showed that BEY could attenuate EAE symptoms via the activation of SCFAs. The influence of BEY on the inflammatory cytokines (IL-17, IL-6, IFN-γ and TGF-β) in MCP-induced EAE mice was further studied (Figure 5B). Unlike TGF-β, IL-17, IL-6 and IFN-γ levels were significantly higher in the EAE group (420 ± 12 pg/mL, 255.1 ± 9.0 and 680 ± 28 pg/mL, respectively) compared to the BEY-administered EAE mouse group (271.1 ±11.3, 199 ± 18 and 490 ± 13.0 pg/mg, respectively) (*p* < 0.02). An insignificant activation of TGF-β was observed in the BEY-treated group (from 181.2 ± 11 to 199 ± 8.0 pg/mL) (*p* < 0.06). Comparable results were obtained for BEY-treated naïve control mice and the untreated groups. These data indicate that BEY displayed a pivotal modulatory role in the CNS of EAE mice. The consistent findings obtained from the positive impacts of BEY on EAE pathogenesis and inflammatory markers led to further study of the cell population of CD4, Th17 and CD8 differentiation (Figure 5C–E). The flow cytometric analysis revealed that BEY treatment led to a significant reduction in the CD4 cell population and the activation of CD8 populations in splenic lymphocytes of MCP-immunized mice (Figure 6A–D).

### 2.6. BEY Alleviates EAE Symptoms via the Regulation of the Transcriptional Factors

**Figure 7:**
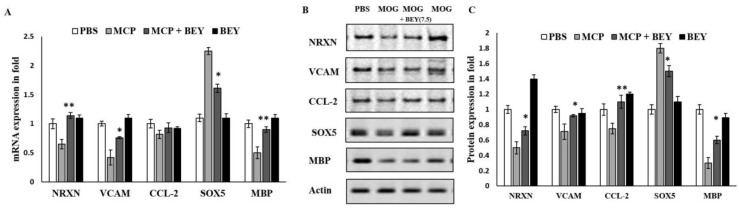
Expression levels of NRXN, VCAM, CCL2, SOX5 and MBP mRNA and protein in MOG-immunized BEY-treated mice. (**A**) mRNAs of NRXN, VCAM, SOX5 and MBP were significantly upregulated in mouse model after BEY treatment. CCL2 was less sensitive in BEY-treated group compared with untreated EAE group. Gapdh was used as internal control to calibrate the expression level. (**B**,**C**) Protein expression was noted in precisely the same manner as mRNA expression. For protein immunoblot studies, actin was used as an internal control. For each group, values were expressed as mean ± SD. For statistical analysis, one-way ANOVA was used, followed by a post hoc *t*-test (significant at * *p* ≤ 0.05, ** *p* ≤ 0.01).

With the exception of CCL2, the mRNA expression levels of NRXN, VCAM, SOX5 and MBP in the microglia of the CNS in BEY-treated EAE mice were significantly upregulated (1.13 ± 0.15-, 0.61 ± 0.04-, 0.93 ± 0.02-, 0.89 ± 0.03-fold) compared to those in MCP-induced mice (0.75 ± 0.1-, 0.43 ± 0.08-, 0.75 ± 0.06-, 0.54 ± 0.05-fold) (Figure 7A). The Western blot results showed a similar confirmatory pattern of protein expression levels of the same transcriptional factors (Figure 7B,C). These findings indicated that BEY treatment of post-immunized mice interacted directly with transcriptional factors that are driven to demyelination and the transmembrane proteins of neurons.

## 3. Discussion

CM fermented by BA showed an increase in total fat and cholesterol as well as a decrease in total carbohydrates, indicating its high sensory quality [12]. Fermented CM has been proven to combat host inflammatory disorders, including IBD and hypercholesterolemia, due to its antioxidant capacity, the protective role of membrane integrity and a balanced immunomodulatory effect [12,35]. In this study, the protective function of BEY against the MCP-induced EAE model of MS was investigated using ex vivo and in vivo approaches.

In inflammatory diseases, CD4 T cells serve as a potential source of proinflammatory cytokines. Depending on the pattern of regulatory transcriptional factors such as SOX5, AHR and NFkB, the polarization of autoimmune diseases progresses in host cell differentiation and maturation [36,37]. The MOG-antigenic stimulation in splenocytes was attenuated by BEY treatment, as evidenced by the substantial suppression of the SOX-5 expression level. Consequently, the inflammatory cytokines (IL-17 and IFN-γ secreted from CD4^+^ Th17 cells) were significantly downregulated upon BEY treatment. These findings point out that BEY has a potential role in signaling CD4 T-cell activation and lineage separation for cytokine secretion in MOG-induced splenocytes [38,39]. TGF-β-, however, was found to be expressed differently in MOG-treated cells, while IL-6 cytokines showed only a minor shift in the BEY-treated group. Conversely, anti-inflammatory responses, defined by the activation of Treg cells in cell polarization in the host, may also be elicited by PBT-fermented products [40].

In this decade, miRNAs—epigenetic non-coding short RNAs—have gained renewed interest as an alternative to traditional genetic determinants. miRNAs are believed to be the molecular targets of autoimmune disorders and can suppress and regulate inflammatory markers [41,42]. In this study, miR218-5p was identified as a SOX5 mRNA target using the target scan tool. BEY exposure positively upregulated miR218-5 expression in spleen lymphocytes in a dose-dependent manner. This was confirmed in this study, since BEY treatment showed reciprocal regulatory effects between miR-218-5p and SOX5. Simpson [43] reported that maternal PBT supplementation influences the level of miR218 and long non-coding RNA in offspring and mediates the gut–placental interaction between the mother and fetus [44]. Our data suggest the potential exploitation of SOX5/miR-218-5p as an effective diagnostic marker for MS. Furthermore, BEY treatment regulated other cytokines and inflammatory markers (IL-17, IL-6, IFNγ and TGF-β) through ex vivo and in vivo experiments. BEY controlled the transcription factor SOX5 based on the role of miR-218, which is an intracellular molecular inhibitor of cell maturation and differentiation.

This study is the first preclinical approach to explore the therapeutic role of BEY in neuronal autoimmune disorders. BEY displayed amelioration of the clinical disease index by improving the recovery of paralysis and demyelination signs in MS mice. These observations were further substantiated by improving body weight and pathophysiology in the host cells. These therapeutic effects of BEY were corroborated by previous evidence using *Lactobacillus reuteri* [45], *Escherichia coli* [46] and combinations of *Lactobacillus* and *Bifidobacteria* spp. [47]. Additionally, BEY showed consistent health-improving anti-inflammatory effects on the colitis mouse model [12].

In the current EAE model, BEY successfully formed the complex of the miR-218/SOX5 axis, leading to the activation of both the Th1 and Treg populations relative to control mice, especially after MCP immunization, which was further confirmed in the histopathological examinations. The inflammatory mediators were reduced, and the graded infiltration of inflamed cells at the site of damage was significantly reduced compared to non-treated mice. Moreover, the intensity of inflammation was proportional to the number of cytokines present in the damaged tissues. Inflammatory markers (IL-17, IL-6, IFNγ and TGF-β) were significantly reduced in BEY-treated MCP-induced mice, suggesting that BEY played a role in attenuating inflammatory cytokines and preserving spinal integrity through the release of SCFA regulatory mediators in the spinal cord of diseased mice. Decisive suppression of inflammatory markers in EAE animals occurs because PBT-mediated SCFAs are an energy source for the mucin layer and also mediate communication between the gut and the brain [47,48,49,50]. Butyric and caproic acids were found to have a gradual rise during BEY therapy of EAE mice compared to the EAE groups, which is consistent with a study that utilized a PBT mixture of Vivomixx (*Lactobacillus* and *Bifidobacterium* spp.) [51].

Numerous types of cells work together to control autoimmune disorders. Some of these cells belong to the IL-17 CD4^+^ group [43,52]. The ameliorating impact of BEY on the myelination of neuronal cells in a CD4-dependent manner is confirmed by the decrease in CD4-positive cells in the BEY-treated mice. The current study showed that the binding of PBT products to these markers causes a significant change. Immunohistologically, EAE mimics MS through Th1 and Th17 immune responses. Conversely, CD4^+^ T cells and Treg cells with the transcription factor FOXP3 play a crucial role in reducing inflammation in the EAE model [53,54]. This research revealed that BEY significantly decreased the CD4, CD8 and TH17 cell populations in the site of neuronal damage and confirmed the alleviation through the inhibition of Th1 and Th17 polarization. These results demonstrate that lymphoid components are expressed differently in various microenvironments in mice that have been vaccinated against the MCP.

Compared to MCP-induced EAE mice, BEY-treated EAE mice exhibited significantly higher mRNA expression levels of NRXN, VCAM, SOX5 and MBP in CNS microglia. These findings were supported by protein expression levels as revealed by Western blotting analysis, providing robustness to the notion that administering BEY to immunocompromised mice induced a direct interaction between BEY and transcriptional factors that drive demyelination and neuronal transmembrane proteins.

There are two therapeutic treatments that use interferons and glatiramer acetate to lessen the inflammation and progress of MS (specifically RRMS) [55,56,57]. However, they have limitations such as side effects, weak adhesion and unfavorable administration. Therefore, PBTs showed a promising and effective therapeutic approach for neurological diseases [58] due to their wide range of healthful benefits, including immunomodulatory effects, gastrointestinal barrier protection, antimicrobial activity and increased mucoadhesion compared to immunosuppressive drugs [59,60,61]. From this study, BEY treatment could be a potential therapeutic approach for neurodegenerative diseases, as has been seen in similar PBT studies previously [61,62].

## 4. Materials and Methods

### 4.1. Animals

For experimental analysis, male C57BL6j mice aged six weeks old and weighing between 18 and 22 g were used. They were obtained from the animal facility at the College of Science, King Faisal University (KFU), Al-Ahsa, Saudi Arabia. Ethical Council of Deanship of Scientific Research guidelines for the use and handling of experimental mice were followed throughout all animal experiments (KFU-REC-2021-OCT-EA00075). The Institutional Animal Care and Use Committee (IACUC) of the King Faisal University authorized all animal studies and care.

### 4.2. BA Growth Conditions and Yogurt Preparation

The PBT bacterial strain (JF836079) used here was characterized earlier for its immunomodulatory and anti-oxidative features. Growth conditions, preservation and yogurt preparation were carried out as in a previous study [63].

### 4.3. In Vitro Stimulation of Splenocytes Using MOG35–55

C57BL6j naïve mice were excised from the spleen to extract the splenocytes using the MACS isolation kit (Miltenyi Biotec, Bergisch Gladach, Germany). The isolated cells were subjected to mechanical dissociation, RBC lysis and 70 µm filtration. The cells were then plated at a density of 10^6^ cells per mL after being suspended in full cRPMI activated with 20 g/mL MOG35–55. After 4h, the cells were treated with BEY (10^6^, 10^7.5^ and 10^9^ CFU/mL of yogurt). Treated cells were evaluated for SOX5 using protein immunoblot [12], splenocyte viability was assessed using SRB toxicity assay and the level of inflammatory cytokines was quantified using ELISA kit [64].

### 4.4. Quantification of Secreted Cytokines by ELISA

The cytokines IL-6, IL-17, IFNγ and TGF-β in the MOG-stimulated cells were quantified according to the Biolegend’s recommendations for quantification [65]. Different samples were measured for relative absorbance and compared to the standard curve, which was determined by the known concentration. Hence, the captured protein concentration contents were defined.

### 4.5. EAE Induction and Clinical Evaluation of Experimental Mice

Mice were divided in a random manner into four groups (six mice in each group), including: group 1: naïve, untreated control mice; group 2: MCP-immunized EAE mice; group 3: MCP was immunized and BEY was administered from day 3 to day 27; group 4: BEY orally administered to mice from day 3 to day 27 of experiment. Mice were immunized with MOG35–55 (100 μg; Peptide International) emulsified in complete Freund’s adjuvant (CFA; Sigma-Aldrich, St Louis, MO, USA) containing 10 mg/mL heat-killed *Mycobacterium tuberculosis* H37Ra (Difco Laboratories, Detroit, MI). The mice were injected intraperitoneally with pertussis toxin (PTX) (List Biological Laboratories, Detroit, MI; 500 ng) on days 0 and 2. A total of 200 µL of emulsion (MCP) was administered subcutaneously on mice’s flanks at four locations. After the 3rd day of immunization, BEY was orally administered at 200 mg/20 g of body weight of mice on every alternate day until sacrifice day (27th day). Mouse models of EAE were evaluated for clinical signs using the following disease score: disease severity is measured on a scale from 0 (healthy) to 4 (death or very sick): decreased tail tone (0.5), impaired righting response (1.5), limp tail and hind limb weakness (2.5), paralysis of both hind limbs (2.5), paralysis of both limbs (2.5), paralysis to the hip (3.5) and death (4.0). The highest number contributed by clinical signs is used in the final count [66].

### 4.6. Histological Analysis

Mice were perfused with 10 mL formalin (10%) and heparinized PBS after being euthanized. The extracted whole brains were then preserved in 70% ethanol and set in paraffin blocks. Next, 4 µm-thick sections were cut and adhered to microscope slides by using a waterbath and a microtome. Slides were deparaffinized with xylene and ethanol washes according to the previously published protocols of Hematoxylin and Eosin (H&E) [67].

### 4.7. Different Inflammatory Markers Assessed in the Spinal Cord

The levels of cytokines (IL-6, IL-17, IFNγ and TGF-β) in the spinal cord of all groups were measured using ELISA kits from Invitrogen, Thermo Fisher Scientific, Vienna, Austria; Cayman, CA, USA, following the guidelines provided by the manufacturer. On an automated ELISA plate reader, the levels of cytokines in the plates were reported at 450 nm (BioTek Instruments, Santa Clara, CA, USA ).

### 4.8. Quantification of SCFAs

The composition of SCFAs was estimated from the cecal content using the previously described methods [68]. In brief, 100 mg of mucus tissue and 400 µL of deionized water were homogenized. Phosphoric acid was used to homogenize the samples, which were centrifuged (12,000× *g* for 15 min) after 30 min. Ethyl butyric acid (Sigma, St Louis, MO, USA) was used as an internal standard to precisely determine experimental concentrations. To each sample, 0.0375 mM caproic acid (Himedia, India) was supplemented. BIOvision chemical kits were used to analyze the samples.

### 4.9. RNA Extraction and Evaluation of Quantitative PCR

The total RNA was obtained from the spinal cord following the trizol extraction method [69]. Exactly 300 ng of RNA was added to prepare cDNA in the Superscript II RT (Invitrogen), and the power SYBR Green Master Mix was used in the amplification of the PCR products using StepOne Real-Time PCR System. Gapdh was used to normalize the samples, and comparative CT method was used to measure the relative mRNA. The primer pairs (Table 1) were used in the expression of specific gene transcripts. To optimize the specificity and sensitivity of the amplification reactions, a melting point analysis was conducted, which was identified with the Sybr Green I dye [70].

### 4.10. Western Blot

Using RIPA lysis buffer, the proximal colons of the mice treated with MCP and BEY were used to produce the protein lysates (Santa Cruz, CA, USA). The lysates were transferred to a PVDF membrane with a thickness of 0.22 m after resolving on an SDS-PAGE gel (10%). The following primary antibodies were used in Western blot assays: NRXN (mouse polyclonal antibody 1:750; Invitrogen, Waltham, MA, USA); VCAM (rabbit polyclonal antibody 1:500; Biorbyt, Cambridge, UK); SOX5 (mouse monoclonal antibody 1:500; Invitrogen, Waltham; CCL2 (mouse monoclonal antibody 1:1000; Invitrogen, Waltham, MA, USA; MBP (rabbit polyclonal antibody 1:1000; Biorbyt, Cambridge, UK); and β-actin rabbit monoclonal antibody 1:1500 (Biorybt, Cambridge, UK).

### 4.11. Statistical Analysis

The Quant Studio software (Applied Biosystems) applied Real-Time PCR data for the analysis with the 2^−ΔΔCT^ method [70]. The Western blot analysis revealed the linear range of the chemiluminescent signals by observing the chemiluminescence of the expressed bands; the densitometry tool in ImageJ software version 1.8 was employed for the quantifications. Data were presented as mean ± SD. For statistical analysis, one-way ANOVA was used, followed by a post hoc *t*-test (significant at *p* ≤ 0.05).

## 5. Conclusions

BEY exhibited promising therapeutic impacts on an autoimmune-mediated neurodegenerative paradigm in MOG-immunized C57BL6j mice, as revealed by robust polyphasic evidence. BEY substantially enhanced neuroprotective markers and reduced the proinflammatory cytokines and demyelination process. BEY acted as a novel orchestrator of SOX5 via the epigenetic factor miR-218, recommending its application as a diagnostic marker for MS. These data pinpoint unequivocally that BEY represents a promising clinical strategy for the therapeutic management of neurodegenerative disorders and could be developed for the promotion of probiotic food as medicine in the future. There are limited studies on probiotics as therapeutics and prevention strategies for neurological disorders. BEY could be assessed in large-scale clinical trials to explore its efficacy against neurodegenerative diseases.

## Figures and Tables

**Table 1:** Primers used to target the mRNA.

Name of the Primer	Forward Primer	Reverse Primer	PCR Product Size (bp)
MBP	ATTCACCGAGGAGAGGCTGGAA	TGTGTGCTTGGAGTCTGTCACC	245
CCL2	GCTACAAGAGGATCACCAGCAG	GTCTGGACCCATTCCTTCTTGG	122
SOX5	CGCCAGATGAAAGAGCAACTCAG	TGAGTCAGGCTCTCCAGTGTTG	147
NRXN	AGGACATTGACCCCTGTGAG	CCTTCATCCCGGTTTCTGTA	241
VCAM	TGA CGA TG CGT GTG CCA GT	GCT GTC GGT TCC CAT TGT CT	228
GAPDH	GTCTCCTCTGACTTCAACAGCG	ACCACCCTGTTGCTGTAGCCAA	188
miR218-5p	CGAGTGCATTTGTGCTTG ATCTA	TGGTGTCGTGGAGTCG	89
U6	CTC GCTTCGGCAGCACA′	AACGCTTCACGAATT TGCGT-	77

## Data Availability

Available from the corresponding author upon request.

## Abbreviations

EAE: Experimental autoimmune encephalomyelitis; MS: Multiple sclerosis; MOG: Myelin oligodendrocyte peptide; CFA: Complete fraud adjuvant; PTX: Pertussis toxin; BA: *Bacillus amyloliquefaciens*; BEY: *Bacillus*-enriched yogurt; IL-6: Interleukin 6; miR: Micro RNA; SOX5: SRY-Box Transcription Factor 5; CM: Camel milk; PBT: Probiotic; IBS: Inflammatory bowel syndrome; GM: Good gut commensal microbiomes; CFU: Colony forming units.
